# Wavelet Analysis and Self-Similarity of Photoplethysmography Signals for HRV Estimation and Quality Assessment

**DOI:** 10.3390/s21206798

**Published:** 2021-10-13

**Authors:** Alexander Neshitov, Konstantin Tyapochkin, Evgeniya Smorodnikova, Pavel Pravdin

**Affiliations:** Welltory Inc., 541 Jefferson, Suite 100, Redwood City, CA 94063, USA; jane@welltory.com (E.S.); pavel@welltory.com (P.P.)

**Keywords:** photoplethysmography, heart rate variability, signal processing, wavelet transform, signal quality

## Abstract

Peak-to-peak intervals in Photoplethysmography (PPG) can be used for heart rate variability (HRV) estimation if the PPG is collected from a healthy person at rest. Many factors, such as a person’s movements or hardware issues, can affect the signal quality and make some parts of the PPG signal unsuitable for reliable peak detection. Therefore, a robust HRV estimation algorithm should not only detect peaks, but also identify corrupted signal parts. We introduce such an algorithm in this paper. It uses continuous wavelet transform (CWT) for peak detection and a combination of features derived from CWT and metrics based on PPG signals’ self-similarity to identify corrupted parts. We tested the algorithm on three different datasets: a newly introduced Welltory-PPG-dataset containing PPG signals collected with smartphones using the Welltory app, and two publicly available PPG datasets: TROIKAand PPG-DaLiA. The algorithm demonstrated good accuracy in peak-to-peak intervals detection and HRV metric estimation.

## 1. Introduction

Heart rate variability (HRV) is the physiological phenomenon of variation in the time interval between heartbeats [1]. Typically, HRV is estimated using electrocardiography (ECG). R peaks, the upward deflections in ventricular depolarization complexes in ECG [2] are detected, and the distances between consecutive R peaks, called RR intervals, are analyzed. There is a substantial body of research that detects R peaks in ECG using various signal processing methods [3], as well as time series classification tools such as interval feature transformation [4].

Photoplethysmography (PPG) is a non-invasive and low-cost optical measurement technique that provides important information about the cardiovascular system [5,6]. PPG tracks blood volume changes in peripheral blood vessels by illuminating the skin and measuring changes in light absorption. In practice, PPG signals are often collected using wrist-worn devices with optical sensors, such as smartwatches [7,8], or using a smartphone camera attached to a user’s finger [9,10] (we will refer to such signals as smartphone PPG). PPG signals are used to estimate heart rate [7,8,10,11], as well as blood oxygen saturation, blood pressure [6,10], etc.

Research [12,13,14,15] shows that, if a PPG signal is collected from a healthy subject at rest, then the intervals between consecutive major peaks in PPG can serve as a substitute for RR intervals in ECG for various heart rate variability metrics. At the same time, PPG signals collected during or right after exercise or from a patient with cardiovascular disease can have peak-to-peak intervals that differ significantly from RR intervals in ECG and cannot be used for HRV estimation [12,15].

**Figure 1:**
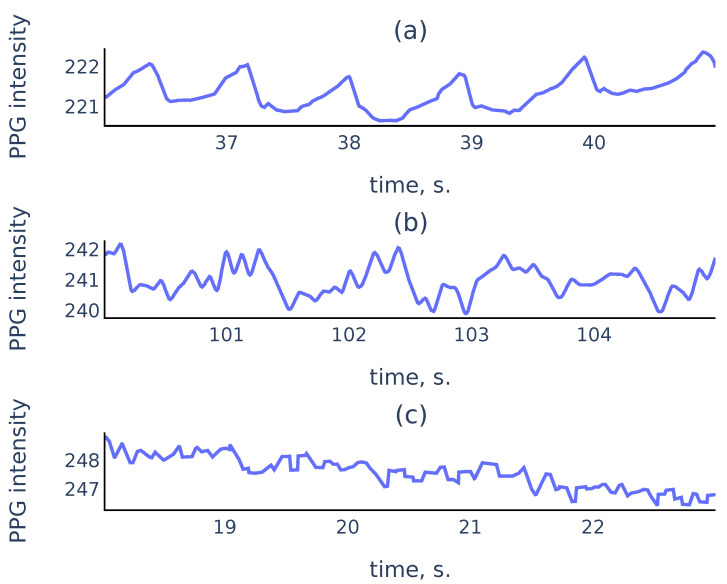
Examples of PPG signals: (**a**) a clean signal with clearly visible peaks; (**b**) a noisy signal where peaks associated to cardiac cycles still can be recognized; (**c**) a corrupted signal where no accurate peak detection is possible.

In practice, PPG signals collected in an uncontrolled environment often suffer from measurement artifacts that can corrupt sufficiently long parts of the signals and make these parts unsuitable for reliable peak detection. Figure 1 shows three common types of smartphone PPG signals that we encounter in practice:

While it is easy to perform well on signals like Figure 1a, a reliable algorithm must also distinguish corrupted parts like Figure 1c to exclude them from the analysis. For the signal parts such as Figure 1b, a reliable algorithm should choose peaks corresponding to cardiac cycles rather than the noise in the signal.

Several algorithms were developed for peak-to-peak interval detection in PPG using slope analysis [16,17], automatic multiscale peak detection [18,19], neural networks [20], adaptive threshold peak detection [21,22]. Existing algorithms filter out erroneous intervals using outlier detection based solely on the lengths of detected intervals. In our algorithm, we propose to analyze the signal during the detected intervals to estimate their reliability. A real-time algorithm for peak detection and signal quality estimation in smartphone PPG was proposed in [9]. As a real-time algorithm, it was restricted in computational complexity and the variety of methods that can be used. Therefore we propose a new offline algorithm that uses continuous wavelet transform (CWT).

CWT is a tool that provides a representation of a signal in the time-scale domain. It is used for time-frequency localization [23] and pattern matching [24]. CWT was successfully used for the analysis of ECG signals [25], electroencephalogram (EEG), and other time series data [26]. Peak detection using continuous or discrete wavelet transform was performed in various contexts in [24,27,28,29,30,31,32].

The main features of CWT that we use in the proposed algorithm are the ridge lines, i.e., curves consisting of points of local maxima at fixed scales in the time-scale domain. Using the correspondence between peaks in the signal and the ridge lines in CWT with the Mexican hat mother wavelet (see Section 2.3), our algorithm uses the ridge lines lengths to detect the peaks in PPG that correspond to the heartbeats. The algorithm also uses the shape of the ridge lines and signal self-similarity characteristics as features to identify corrupted parts in PPG signals. We describe the proposed algorithm in detail in Section 3.

We evaluate the accuracy of the proposed algorithm on three different publicly available datasets: Welltory-PPG-dataset, TROIKA [7] and PPG-DaLiA [8]. Note that, since PPG signals during exercise cannot be used for HRV estimation according to [12,15], for validation using TROIKA [7] and PPG-DaLiA [8] we only used parts of the signals that were collected before any labeled physical activity. Welltory-PPG-dataset is a new publicly available dataset that we introduce in this paper. It consists of smartphone PPG measurements with simultaneously collected ground truth RR intervals from the Polar H10 chest strap device. We introduce this dataset, since most publicly available PPG datasets (such as TROIKA [7] and PPG-DaLiA [8]) are collected using wrist-worn devices and are aimed at heart rate detection during physical activity, and the existing smartphone PPG dataset [33] consists of 10-second signals which are too short for a reliable HRV assessment. We describe the dataset structure and data collection process in Section 2.1.1.

While CWT is used for various signal types, including ECG, EEG, etc., it has not been used before for peak detection in PPG. In contrast with existing PPG analysis algorithms, our algorithm filters out unreliable peak-to-peak intervals by detecting corrupted parts in the signal. Our algorithm demonstrated good accuracy in basic HRV metrics on three publicly available datasets containing PPG from different sources. Thus, we conclude that the algorithm should generalize well to various PPG signals of different origins, be robust to signal corruption, and can be able to be used for reliable HRV estimation from PPG signals collected in an uncontrolled environment.

The paper is organized as follows. The datasets used for algorithm validation are described in Section 2.1. HRV metrics used for accuracy estimation are described in Section 2.2. Section 2.3 contains a discussion about CWT and the ridge lines. A detailed description of the proposed algorithm is given in Section 3. Section 4 contains tables with HRV metrics estimation errors on the used validation datasets. Section 5 provides additional discussion. It contains a justification of the methods used in the proposed algorithm, its limitations, and its comparison with previously developed algorithms. Moreover, it contains a justification of the choice of the ground truth labels in Welltory-PPG-dataset. Section 6 contains concluding remarks.

## 2. Materials and Methods

### 2.1. Datasets

#### 2.1.1. Welltory-PPG-Dataset

This is a newly introduced dataset. It is publicly available at https://github.com/Welltory/welltory-ppg-dataset (accessed on 1 July 2021). The dataset consists of 21 records containing three time series: red, green, and blue channel PPG signals, and simultaneously collected RR intervals. RR intervals were collected using a Polar H10 chest strap and manually examined by an expert to ensure their accuracy. PPG data were obtained via a smartphone camera using the Welltory app. Signal lengths vary from 68 to 112 s.

##### Data Collection

Each participant put their index finger over a smartphone camera, which recorded a video. For each frame in the video stream, we record its capture time and three numbers: r, g, b, which are the average values of red, green, and blue components of the frame pixel colors. Each participant was simultaneously wearing the Polar H10 chest strap. After each measurement was complete, the RR intervals detected by the Polar device during the measurement were collected. Each dataset record consists of the following arrays:

- Time: moments of camera frame capture times in milliseconds elapsed from the measurement start;
- R, G, B: arrays of numbers r, g, b for all captured frames;
- RR: sequence of RR intervals collected from the Polar device during the measurement.

##### Participants

Thirteen healthy volunteers between the ages of 25 and 35 participated in the data collection. Each participant made one or two measurements using their Android smartphone. All participants provided written informed consent for publication.

#### 2.1.2. Previously Published PPG Datasets

In addition to Welltory-PPG-dataset, we used two publicly available datasets: TROIKA [7] and PPG-DaLiA [8] for algorithm validation. These datasets were introduced for heart rate estimation and contain simultaneous PPG and ECG signals and accelerometer readings collected during exercise and other labeled physical activity. Since PPG signals collected during exercise are unsuitable for HRV estimation [12,15], we used signal parts not labeled with physical activity. In TROIKA these parts are the initial 30-second chunks of the signals. In PPG-DaLiA these are the intervals that are labeled as “sitting”.

### 2.2. HRV Metrics

We use two basic metrics of heart rate variability: SDNN, and RMSSD. For a sequence of normal RR intervals $rr_{1},\ldots ,rr_{n}$, its SDNN is defined as the standard deviation:

(1) $$\mathrm{SDNN}\left(rr_{1},\ldots ,rr_{n}\right)=\sqrt{\frac{1}{n}\sum_{i=1}^{n}{\left(rr_{i}-\bar{rr}\right)}^{2}},\;\bar{rr}=\frac{1}{n}\sum_{i=1}^{n}rr_{i}.$$

RMSSD, the root mean square of the successive differences of RR intervals, is defined as

(2) $$\mathrm{RMSSD}\left(rr_{1},\ldots ,rr_{n}\right)=\sqrt{\frac{1}{n-1}\sum_{i=1}^{n-1}{\left(rr_{i+1}-rr_{i}\right)}^{2}}.$$

The latter metric has a geometric interpretation in terms of the Poincaré plot of the sequence $rr_{1},\ldots ,rr_{n}$: for the distribution of pairs $\left(rr_{i},rr_{i+1}\right)$ of consecutive RR intervals, RMSSD is proportional to the standard deviation of the distance from the points $\left(x,y\right)=\left(rr_{i},rr_{i+1}\right)$ to the main diagonal x=y.

### 2.3. Continuous Wavelet Transform

For a mother wavelet function ψ(t) let $\psi_{\left(a,b\right)}$ denote its scaled and translated version:

(3) $$\psi_{a,b}\left(t\right)=\frac{1}{\sqrt{a}}\psi \left(\frac{t-b}{a}\right)$$

Here a>0 is the scale parameter, and b∈R is the translation parameter. Then for a function x(t),t∈R its continuous wavelet transform (CWT) is defined as [23] ([2.4]):

(4) $$X_{w}\left(a,b\right)=\int_{R}x\left(t\right)\psi_{a,b}\left(t\right)dt$$

The coefficients $X_{w}\left(a,b\right)$ show matching between the signal x(t) and the mother wavelet ψ dilated with scale *a* and centered at position *b*. In our algorithm we chose ψ to be the Mexican hat wavelet function. It is proportional to the second derivative of the Gaussian function and is defined as:

(5) $$\psi \left(t\right)=\frac{2}{\sqrt{3}\pi^{1/4}}\left(1-t^{2}\right)e^{-t^{2}/2}$$

**Figure 2:**
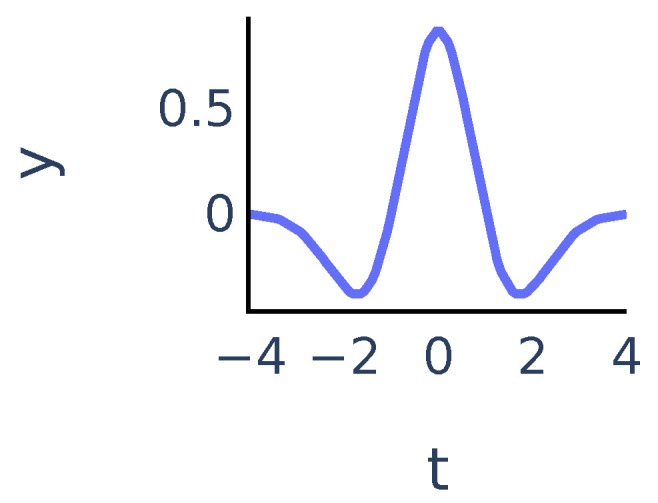
Mexican hat wavelet function y=ψ(t).

It exhibits typical features of a single peak (Figure 2):

We will say that (a,b) is a ridge point if the function $t\mapsto X_{w}\left(a,t\right)$ has a local maximum at point t=b. For a fixed *a* the ridge points at scale *a* show the time instants where the signal x(t) most resembles the peak at scale *a*. We will consider continuous curves in the (a,b)-plane that consist of ridge points. Following [24], we will call such curves ridge lines. So ridge lines indicate the position of peaks in the signal at different scales. We discuss this in more detail in Section 3.3.1.

## 3. Proposed Algorithm

In this section, we describe the proposed algorithm. In the rest of the paper, we will refer to peaks in PPG that correspond to heartbeats as R peaks for brevity. Peak-to-peak intervals will be referred to as RR intervals. We will use the CWT of the PPG signal with the Mexican hat function as the mother wavelet as the main tool.

In the first step of the algorithm, we perform signal preprocessing that addresses the two most common problems: abrupt shifts, or steps in signals and signals becoming almost constant due to hardware issues. A detailed description of preprocessing steps is given in Section 3.1. Then we perform R peak detection. To detect R peaks in the signal, we will identify their corresponding ridge lines in the CWT. Our algorithm for R peak detection is based on two principles that must hold for non-corrupted PPG signals collected from healthy subjects at rest:

- the R peaks correspond to longer ridge lines, i.e., lines that are present on a larger scale range (see Section 3.3 for more details).
- PPG signals are almost periodic, so normal R peaks arise approximately at a frequency determined by the heart rate that varies gradually over time.

Therefore, we use a 2-step process for R peak detection. First, we estimate the heart rate from the PPG signal using the short-time Fourier transform spectrogram of the signal. Then, the algorithm uses the estimated heart rate as additional information to choose ridge lines corresponding to R peaks. Informally speaking, the algorithm chooses the most persistent ridge lines that arise in the CWT at a frequency corresponding to the estimated heart rate. Finally, the locations of R peaks are predicted as positions of the chosen ridge lines at the smallest scale. A detailed description of the R peak detection algorithm is given in Section 3.3.

After the R peaks are detected, we consider the corresponding RR intervals. We evaluate the quality of detected RR intervals to identify and discard RR intervals found in corrupted parts of the signal. Since a non-corrupted PPG signal is almost periodic, the quality estimation is based on two considerations:

- A signal must have similar shapes inside detected RR intervals;
- The ridge lines in CWT that define the edges of a RR interval must have similar shapes. In particular, the distances between them should be approximately the same on different scale levels.

We assign to each RR interval its quality score based on the principles above and then discard the RR intervals with quality scores below an automatically determined threshold. We give a detailed description of the quality score calculation and the choice of the quality threshold in Section 3.4.

**Figure 3:**
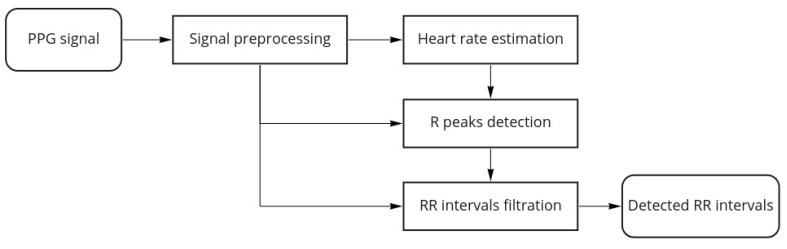
Algorithm workflow.

Figure 3 shows the proposed algorithm workflow for a single channel PPG:

Once the algorithm finishes, we estimate the overall quality of the signal as the following ratio:

(6) $$\mathrm{discarded}\;\mathrm{ratio}=\frac{n_{discarded}}{n_{total}}$$

where $n_{total}$ is the total number of RR intervals detected by the R peaks detection part of the algorithm and $n_{discarded}$ is the number of detected RR intervals that were discarded by the filtration part of the algorithm.

In some cases, valleys in the PPG signals can be easier to locate than peaks. Thus, as a final step, we apply our algorithm to both the PPG signal and the negative of the PPG signal and obtain two sets of detected RR intervals. Then we choose the result that has the smallest discarded ratio as the algorithm output.

For a multi-channel PPG signal (e.g., smartphone PPG containing values in the red, green, and blue channels of the video frames captured by a smartphone camera), we choose the best channel as the one that has the smallest discarded ratio. Note that research [34] suggests that channels with shorter wavelength should have the best signal to motion ratio. We do not make an a priori choice of the channel to avoid issues with device-specific color representations.

### 3.1. Signal Preprocessing

Most smartphone cameras provide frames at a rate of 30 frames per second. To increase accuracy, we interpolate the signal to a uniformly sampled sequence with a sampling frequency f=100 Hz, as the upsampling is necessary for accurate HRV estimation [17]. Most cameras produce red, green, and blue channel values in the standard range (0, 255). If the PPG signal is given in another range, we rescale it to the standard range. Signal preprocessing aims to identify the following common issues with the signal:

- Step detection. In smartphone PPG signals, removing from or reapplying the finger to the camera results in abrupt steps in the signal. To detect such steps, we compute the running amplitude with a window length of 1 s. If the running amplitude exceeds 4 times the median of the running amplitude, a step in the signal is detected. The threshold value 4 was chosen empirically by examining a number of examples.
- Constant signal detection. Sometimes the signal becomes constant if there are issues with color rendering in the frame or there is no finger over the camera at all. Analysis of examples shows that 0.1 is a reliable threshold value for running signal amplitude to detect a constant signal.

Parts of the signal labeled as having signal steps or constant signal are set to zero. Then, we split the signal into continuous chunks between the labeled parts and set the chunks shorter than 2 s to zero. For every remaining chunk, we remove the trend by subtracting the running average in 2 s windows and apply a low-pass filter with a 10 Hz threshold to avoid aliasing and remove high-frequency noise.

### 3.2. Heart Rate Estimation

In this subsection, we describe the algorithm for heart rate estimation from a PPG signal. Our approach is similar to the TROIKA [7] in that we are estimating heart rate by constructing a continuous curve consisting of peaks in the spectrogram columns. Our context is different from one in [7] in two aspects: firstly, we are interested in PPG collected at rest, so we expect motion artifacts associated with occasional movement, rather than intensive physical exercise considered in [7]. Secondly, we do not collect accelerometer data to estimate the subject’s movements. Therefore we use the following approach:

- First we filter the spectrogram using a convolution with a 2d filter that highlights curves with a bounded rate of change;
- We consider local maxima in the columns of the filtered spectrogram;
- We find a rough estimate of heart rate frequency and construct a continuous curve consisting of local maxima that are closest to the estimate.

We describe our approach in detail below. Suppose that $x_{n}$ is a discrete signal obtained by a uniform sampling with frequency *f* from a function x(t) with a discrete step Δt:

(7) $$x_{n}=x\left(t_{n}\right),\;t_{n}=n\Delta t,\;\Delta t=1/f,\;n=1,2,\ldots N$$

#### 3.2.1. Sliding Window Spectrogram

As a feature, we use the signal spectrogram computed with a sliding Dirichlet window of 5 s in length. The spectrogram is computed as the squared magnitude of the short time Fourier transform (STFT) for frequencies $f_{k}$, k=1,…80 uniformly sampled between 0.5 and 3.3 Hz, with stride = 0.5 s:

(8) $$Spectrogram\left[k,j\right]={\left|\sum_{n=s_{j}}^{s_{j}+5f}x_{n}e^{-2\pi if_{k}t_{n}}\Delta t\right|}^{2},\;s_{j}=0.5*f*j$$

**Figure 4:**
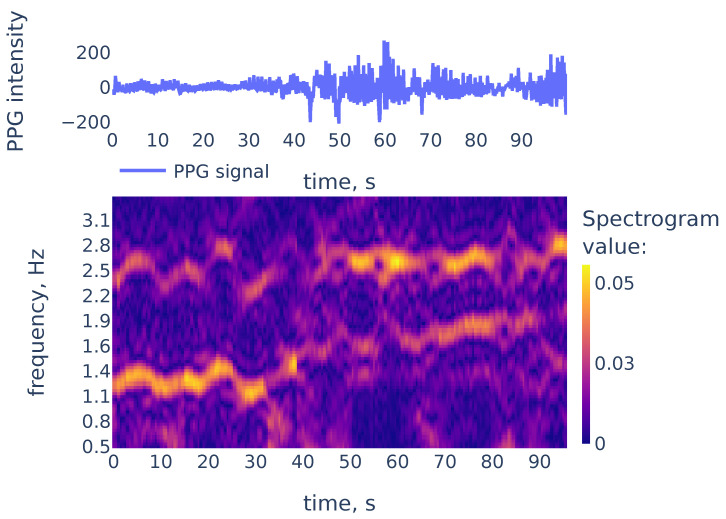
The PPG signal (**top**) and its corresponding STFT spectrogram (**bottom**), part of the PPG signal of subject 01 in the TROIKA dataset. Heart rate frequency is growing from 1.2 Hz to 1.9 Hz.

While peaks in a spectrum of a single window may be associated with measurement artifacts, the heart rate corresponds to a continuous curve in the spectrogram. An example of the spectrogram is given in Figure 4.

**Figure 5:**
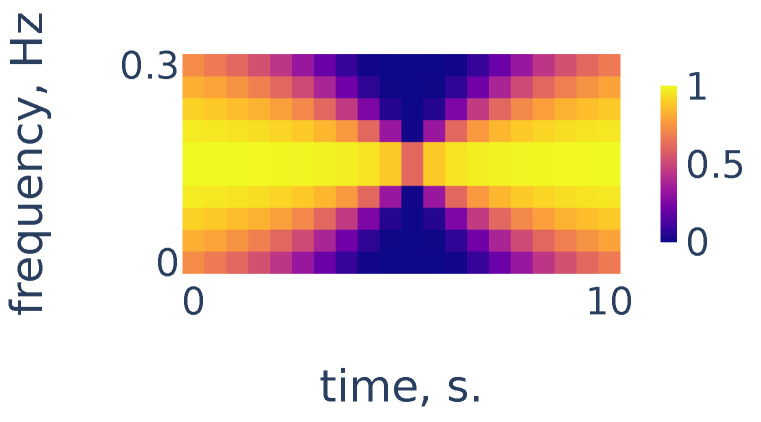
Continuity filter highlighting curves with bounded rates of change; the horizontal length is adjusted to correspond to 10 s of time and the vertical length corresponds to a change in frequency of 0.3 Hz.

As we can see in Figure 4, the heart rate frequency in that example gradually grows from 1.2 Hz at the beginning to 1.9 Hz at the end. The curve is clearly seen for the first 30 s and, after that, it becomes smudged between 30 and 50. To simplify the curve detection we convolve the spectrogram with a 2d filter shown in Figure 5, which highlights continuously evolving curves on the spectrogram:

#### 3.2.2. Local Maxima in the Spectrogram Columns

After the filtration, let us consider the points of local maxima in the columns of the spectrogram. We will construct the curve associated with heart rate frequency by following these points of local maxima in a continuous manner. To find the initial point on the curve, we need to choose between the local maxima in the first column of the spectrogram. In some cases, the point of the highest local maximum does not necessarily correspond to the actual heart rate. Therefore, we find a rough estimate of the heart rate and then choose the peak that is closest to this estimate. To find a rough estimate, we consider a bandpass filtration of the PPG signal with a narrow bandwidth and count the number of peaks and the number of zero crossings in the filtration. For more in detail, we use Algorithm 1. It takes *i*, an index of the spectrogram column as an input, and returns the frequency of a peak in the *i*-th spectrogram column, such that this frequency is the closest to the rough heart rate estimate.
**Algorithm 1** Rough estimate of heart rate frequency in the *i*-th spectrogram column1:s=i-th column in spectrogram2:t_start=0.5*i     ▹ start time of the *i*-th sliding window with stride 0.5 s3:test_len=min(10,signalduration−t_start)4:x= part of the signal between t_start and t_start+test_len seconds5:$f_{max}=$ frequency corresponding to the maximum value of *s*6:**if**$f_{max}>2$**then**7:    threshold=2.58:**else if**$f_{max}<1$**then**9:    threshold=1.510:**else**11:    threshold=212:**end if**13:$x_{sm}=$ bandpass filtration of *x* with cutoff frequencies 0.1 and threshold14:n_zero_cross= number of times $x_{sm}$ crosses the zero level15:n_loc_max= number of local maxima in $x_{sm}$16:upper_estimate=1/test_len*n_loc_max17:lower_estimate=1/test_len*0.5*n_zero_cross18:estimate=0.5*(lower_estimate+upper_estimate)19:F= frequencies corresponding to 3 highest peaks in *s*20:$f_{hr}={arg min}_{f\in F}\left|f-estimate\right|$  **return** $f_{hr}$

Now we construct a curve in the spectrogram plane showing heart rate frequency during the measurement. First, we choose the initial frequency using Algorithm 1 for the 0-th column of the spectrogram. Then we construct the continuous curve following along local maxima in columns of the spectrogram. If at some step we cannot proceed continuously, then we perform Algorithm 1 again to find the point of local maximum in the spectrogram column that is closest to the rough estimate. Along the way, we apply exponential smoothing to the obtained curve for a more robust estimate. In more detail, we use the Algorithm 2:
**Algorithm 2** Construction of the heart rate frequency curve1:n= number of columns in spectrogram2:hrf[0]= output of Algorithm 1 for the 0-th column3:**for***i* in range(1, n) **do**4:    F= frequencies corresponding to 3 largest local maxima in the *i*-th column of spectrogram5:    $f_{cl}={arg min}_{f\in F}\left|f-\mathrm{hrf}\left[i-1\right]\right|$6:    **if** $|f_{cl}-\mathrm{hrf}\left[i-1\right]|<0.1$ **then**7:        $\mathrm{hrf}\left[i\right]=f_{cl}$8:    **else**9:        $f_{next}=$ output of Algorithm 1 for the *i*-th column10:        $\mathrm{hrf}\left[i\right]=0.95*\mathrm{hrf}\left[i-1\right]+0.05*f_{next}$11:    **end if**12:**end for**

**Figure 6:**
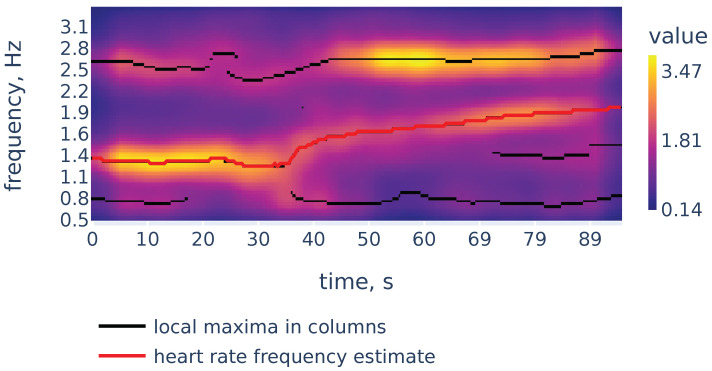
Filtered spectrogram. Black lines show the curves consisting of local maxima in the spectrogram columns.

Now let us demonstrate the work of Algorithm 2. Figure 6 shows the filtered version of the spectrogram of Figure 4, the curves consisting of local maxima in the columns, and the heart rate frequency curve constructed by Algorithm 2. It correctly identifies the heart rate frequency change during the measurement.

### 3.3. R Peak Detection

#### 3.3.1. Signal Scalogram and Ridge Lines

Recall our notation for the signal $x_{n}$ that is uniformly sampled from function x(t) with frequency *f*:

$$x_{n}=x\left(t_{n}\right),\;t_{n}=n\Delta t,\;\Delta t=1/f,\;n=1,2,\ldots N$$

where variable *t* represents time measured in seconds. Let ψ denote the Mexican hat wavelet function given from Equation (5). Consider scales $a_{k}$ evenly spaced on a logarithmic scale:

(9) $$a_{k}=0.05*{\left(1.0376\right)}^{k-1},\;k=1,\ldots ,50$$

Define the scalogram of the signal $x_{n}$ as the finite sum approximation to the integral that defines CWT of the function x(t) in Equation (4):

(10) $$Scalogram\left[k,n\right]=\sum_{m=1}^{N}x_{m}\psi_{a_{k},t_{n}}\left(t_{m}\right)\Delta t,\;n=1,\ldots ,N,\;k=1,\ldots ,50$$

The chosen scales range from $a_{1}=0.05$ to $a_{50}=0.3.$ This range was chosen empirically to accurately reflect the position of R peaks on the smallest scale and provide enough smoothing to remove noisy peaks on the large scale.

**Figure 7:**
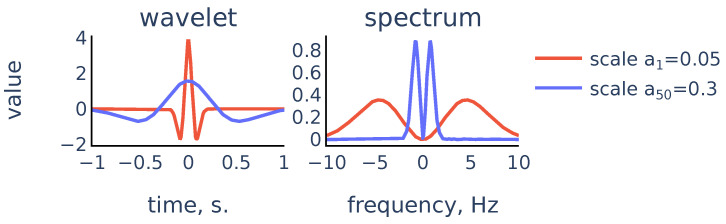
Scaled Mexican hat wavelet and its frequency spectrum for the smallest scale $a_{1}$ and the largest scale $a_{50}$ in the chosen scale range.

**Figure 8:**
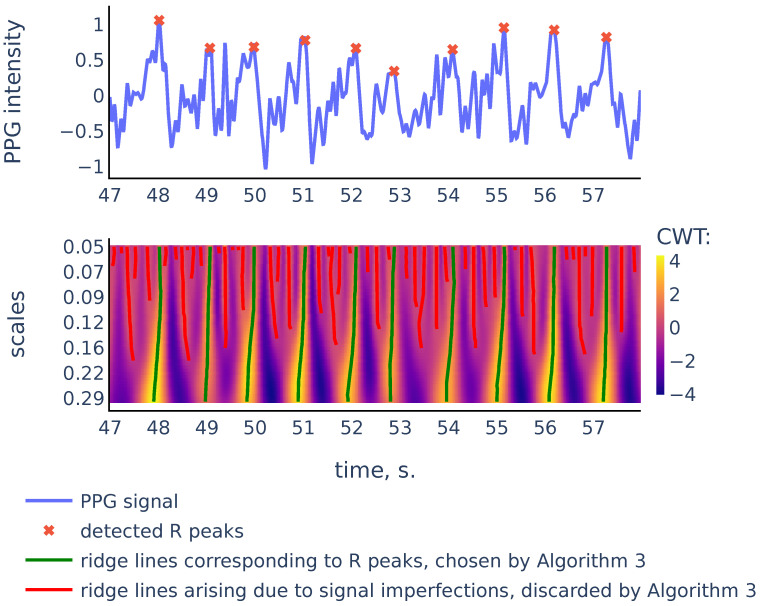
PPG signal, the corresponding scalogram computed with the Mexican hat wavelet for the scale range $a_{1},\ldots a_{50}$, and the ridge lines in the scalogram. This signal is a part of the red channel PPG from subject 01 in the Welltory-PPG-dataset. The R peaks in the signal are detected as top points of the ridge lines chosen by the filtration Algorithm 3.

So the scalogram is a discretization of the CWT, thus the rows of the scalogram represent similarities of the signal with a typical peak shape on the corresponding scales. The ridge lines in CWT will correspond to ridge lines in the (k,n) plane consisting of points of local maxima in the scalogram rows Scalogram[k,:] Note that convolution with the scaled wavelet performs bandpass filtration, with wider bandwidth for smaller scales and narrower bandwidth for larger scales, as we can see in Figure 7. Thus, we can consider rows of the scalogram as filtration, or smoothing of the initial signal, and the ridge lines indicate locations of peaks in the signal at different levels of smoothing. The R peaks in the signal are the more persistent peaks, which exist at different levels of smoothing, and therefore R peaks are tips of longer ridge lines that are present on a larger scale range. Figure 8 shows a typical example that illustrates this idea:

As we can see in Figure 8, there are more ridge lines in the upper part of the scalogram, corresponding to noisy peaks in the signal, but we are able to distinguish ridge lines that correspond to R-peaks by choosing the longer lines and using our previous estimates of heart rate as a heuristic that suggests how often the actual ridge lines are expected to appear on the scalogram plane. In the next section we will discuss in detail the Algorithm 3 that chooses the ridge lines that correspond to R peaks.

#### 3.3.2. Choosing Ridge Lines Associated to R-Peaks

Recall that we defined scalogram in Equation (10) as a matrix of shape (50,N) For a ridge line curve and a scale $a_{k},k=1,\ldots ,50$ denote by $curve[a_{k}]$ the time coordinate of the point on curve with scale level $a_{k}$ (if such a point exists):

For a given set of ridge lines $curve_{1},\ldots ,curve_{m}$ sorted according to their top points $curve_{i}\left[a_{1}\right]$ define their RR intervals as the distances between the top points:

(11) $$rr_{i}=curve_{i+1}\left[a_{1}\right]-curve_{i}\left[a_{1}\right]$$

We will use these RR intervals to determine which curves to choose. First, we choose all the ridge lines that stretch from the bottom to the top of the scalogram. These are the curves corresponding to the most persistent peaks in the signal that are likely to be *R*-peaks. Some of the remaining curves are added to the set of chosen curves based on the likelihood of observing the corresponding set of RR intervals given the previously estimated heart rate frequency.

We estimate the likelihood of a set of potential RR intervals as follows. According to [35], the length of RR intervals can be modeled as Inverse Gaussian distribution. In practice, the Inverse Gaussian distribution can be approximated by log-normal distribution [36], thus we may assume that logarithms of RR intervals are normally distributed, so the average log-likelihood of a set of RR intervals given the heart rate frequency is proportional to

(12) $$estimated-likelihood\left(rr,hrf\right)=-\frac{1}{k}\sum_{i=1}^{k}{\left(\log\left(rr_{i}\right)-\log\left(expected\_rr_{i}\right)\right)}^{2}$$

where $hrf=(hrf_{1},\ldots ,hrf_{k})$ and $hrf_{i}$ the expected heart rate frequency during interval $rr_{i}$, and $expected\_rr_{i}=1/hrf_{i}$ is the expected length of RR interval. We use this estimate of likelihood to finally choose the ridge lines associated to R-peaks, as described in Algorithm 3:
**Algorithm 3** Choosing ridge lines in the scalogram1:hr= estimated heart rate frequency given by Algorithm 22:chosen_curves= ridge lines with lowest point below the scale level 0.244 and highest point over the scale level 0.0693:candidate_curves= ridge lines with lowest point between the scale 0.244 and 0.069 and highest point over the scale level 0.069.4:Sort candidate_curves by the height of the lowest point in the increasing order5:**for**curve in candidate_curves **do**6:    proposed_set=chosen_curves∪{curve}7:    rr_current= set of intervals between curves from chosen_curves8:    rr_proposed = set of intervals between curves from proposed_set9:    current_likelihood = estimated-likelihood(rr_current,hr)10:    proposed_likelihood = estimated-likelihood(rr_proposed,hr)11:    **if** proposed_likelihood>current_likelihood **then**12:        chosen_curves=proposed_set13:    **end if**14:**end for**15:**return**chosen_curves

### 3.4. RR Intervals Filtration

PPG signals often contain corrupted parts where no accurate R-peak detection is possible, so these parts must be discarded to ensure robust HRV estimation. To identify such parts we use the following method. After we have detected RR intervals, we assign to each detected interval a number between 0 and 1 (its quality) that reflects the likelihood that the found interval is accurate and reliable. Then a quality threshold is determined and the RR intervals with the quality below the threshold are discarded.

To develop a quality estimate, we use the following principles:

- Parts of the signal inside the neighbor intervals must have similar shapes and amplitudes;
- The distance between ridge lines defining the neighbor *R*-peaks must be approximately the same on different resolution levels.

**Figure 9:**
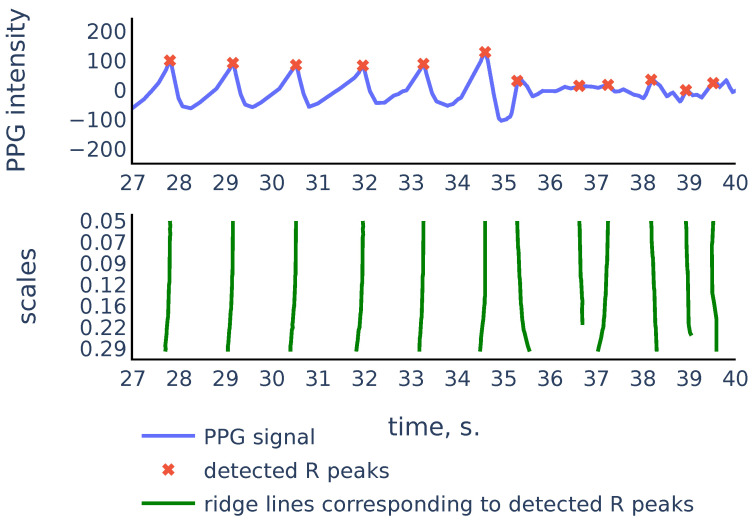
Part of the S3 signal of the PPG-DaLiA dataset.

**Figure 10:**
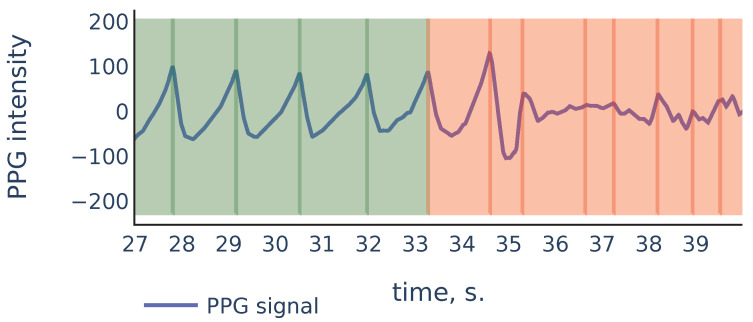
Filtration of the RR intervals of Figure 9. Green intervals are kept by the algorithm and red intervals are discarded.

Let us consider the example shown in Figure 9 to illustrate the principles above:

As we can see, in the corrupted part of the signal starting from 35 s, the signal parts inside RR intervals often can be weakly correlated, their amplitudes can be significantly different, and also the corresponding ridge lines do not repeat the same shape, and they bend in different ways, so the distance between varies on different scale levels more than it does for ridge lines in non-corrupted part of the signal. In the following discussion, we will describe our algorithm that filters out the RR intervals in the corrupted parts of the signal. For the signal shown in Figure 9 it produces the result shown in Figure 10:

To define the quality of the RR intervals, we introduce two auxiliary quality measures: similarity-based quality and CWT-based quality. In the following discussion, we denote by σ(x)=1/(1+exp(−x)) the sigmoid function.

#### 3.4.1. Similarity-Based Quality

Let $x=(x_{1},\ldots ,x_{n})$ and $y=(y_{1},\ldots ,y_{k})$ be two signal chunks. Let $x_{r}$ and $y_{r}$ denote *x* and *y* resampled to vectors of size 50. Define the correlation similarity between *x* and *y* as the correlation coefficient between $x_{r}$ and $y_{r}$, limited by 0 from below:

(13) $$s_{corr}\left(x,y\right)=\max\left(corr\left(x_{r},y_{r}\right),0\right)$$

Let amp(x)=max(x)−min(x) and amp(y)=max(y)−min(y) denote the amplitudes of signals *x* and *y*. Suppose that amp(x) is greater than amp(y) and denote their quotient by $r_{amp}=amp\left(x\right)/amp\left(y\right)$. Define the amplitude similarity as

(14) $$s_{amp}\left(x,y\right)=\sigma \left(2\left(3.5-r_{amp}\right)\right)$$

The formula (14) is chosen empirically to assign low values to pairs of signals where amplitudes are different by 4 times and more. Finally, define the similarity score between *x* and *y* as the geometric mean of its correlation similarity and amplitude similarity:

(15) $$s_{sim}\left(x,y\right)=\sqrt{s_{corr}\left(x,y\right)s_{amp}\left(x,y\right)}$$

Now, if $rr_{1},\ldots ,rr_{n}$ is a sequence of detected RR intervals with R-peaks located at indices $k_{1},\ldots ,k_{n+1}$, then the similarity quality of the *i*-th RR interval $rr_{i}$ is defined as the geometric mean of its similarities with the neighbor RR intervals (using a single neighbor for i=1 or i=n):

(16) $$q_{sim}\left(rr_{i}\right)=\sqrt{s_{i}s_{i+1}},\;s_{i}=s_{sim}\left(x\left[k_{i-1}:k_{i}\right],x\left[k_{i}:k_{i+1}\right]\right),\;i=2,\ldots n-1$$

#### 3.4.2. CWT-Based Quality

Suppose that $curve_{1}$ and $curve_{2}$ are two ridge lines. Consider a sequence $rr\left[f_{k}\right]=\left|curve_{1}\left[f_{k}\right]-curve_{2}\left[f_{k}\right]\right|$ for all 1⩽k⩽50 where both $curve_{1}\left[f_{k}\right]$ and $curve_{2}\left[f_{k}\right]$ are defined. Let

(17) $$rr_{std}\left(curve_{1},curve_{2}\right)=\mathrm{std}\left(rr\left[f_{k}\right]\right)/f*1000$$

be the standard deviation of distances between points on $curve_{1}$ and $curve_{2}$ measured in milliseconds. Now suppose that ends of some $rr_{i}$ intervals were detected using curves $curve_{1}$ and $curve_{2}$ in the scalogram. Then we define the CWT-based quality of the interval $rr_{i}$ as

(18) $$q_{CWT}\left(rr_{i}\right)=\sigma \left(\left(rr_{std}\left(curve_{1},curve_{2}\right)-50\right)/5\right)$$

#### 3.4.3. Filtration

Suppose $rr_{1},\ldots ,rr_{n}$ is the sequence of detected RR intervals. Define the quality of each of the RR intervals as the product of similarity- and CWT-based quality:

(19) $$q\left(rr_{i}\right)=q_{sim}\left(rr_{i}\right)q_{CWT}\left(rr_{i}\right)$$

Now we filter intervals by quality using an automatically determined threshold as follows:

- Select all intervals $rr_{i}$ with quality $q(rr_{i})$ greater than 0.8
- Sort the qualities of the selected intervals in descending order. Denote the resulting sequence by
  $$q_{1}^{\prime }⩾q_{2}^{\prime }⩾\ldots ⩾q_{n^{\prime }}^{\prime }$$
- Set the quality threshold as $q_{i_{0}}^{\prime }$ where the index $i_{0}$ maximizes the product $iq_{i}^{\prime }:$
  (20) $$q_{cutoff}=q_{i_{0}}^{\prime },\;i_{0}=\underset{i=1,\ldots ,n^{\prime }}{arg max}iq_{i}^{\prime }$$

The initial rigid threshold of 0.8 is chosen empirically based on a number of signals examined. The final choice of the threshold value allows us to cut off the intervals that have substantially smaller quality than the rest of the intervals while trying to maximize the number of remaining intervals.

#### 3.4.4. Outlier Detection

In addition to quality-based filtration, we apply outlier detection in the sequence of RR intervals based on their lengths. We use the following algorithm similar to one introduced in [37]. Suppose that $rr_{1},\ldots ,rr_{n}$ is the sequence of detected RR intervals lengths. We then mark certain RR intervals as outliers and discard them from the final answer using Algorithm 4:
**Algorithm 4** Algorithm to check if the *i*-th interval in $rr_{1},\ldots ,rr_{n}$ is an outlier1:$p_{10},m,p_{90}$ = 10-, 50-, and 90-percentile of $\left[rr_{i-13},\ldots rr_{i+13}\right]$2:$rr_{max}=max\left(rr_{i},rr_{i+1}\right)$3:$rr_{min}=min\left(rr_{i},rr_{i+1}\right)$4:**if**$\left(rr_{min}<min\left(m-50,p_{10}-0.2*amp\right)\right)$5:   and $\left(rr_{max}>max\left(m+50,p_{90}+0.2*amp\right)\right)$6:   and $\left(p_{10}<\left(rr_{max}+rr_{min}\right)/2<p_{90}\right)$ **then**7:    mark $rr_{i}$ and $rr_{i+1}$ as outliers8:**end if**9:**if**$rr_{i}<m*0.7$ and $rr_{i+1}<m*0.7$ and $p_{10}<rr_{i}+rr_{i+1}<p_{90}$ **then**10:    mark $rr_{i}$ and $rr_{i+1}$ as outliers11:**end if**12:**if**$rr_{i}>1.6*m$**then**13:    mark $rr_{i}$ as an outlier14:**end if**15:**if**$rr_{i}<min\left(0.7*m,p_{10}\right)$**then**16:    mark $rr_{i}$ as an outlier17:**end if**

## 4. Results

We tested our algorithm’s accuracy on three datasets: Welltory-PPG-dataset, TROIKA, and PPG-DaLiA, using two HRV metrics: SDNN defined by Equation (1) and RMSSD defined by Equation (2). For each PPG signal and a sequence of reference RR intervals, we compute the following quantities:

- Discarded ratio: ratio of the number of RR intervals that were discarded by the filtration part of the algorithm to the total number of RR intervals detected by the algorithm peak detection part, cf. Equation (6).
- SDNN ae (RMSSD ae): the absolute error of estimation of SDNN (RMSSD), i.e., the difference between SDNN (RMSSD) derived from the sequence of intervals detected by the algorithm in PPG, and SDNN (RMSSD) derived from the sequence of reference RR intervals.
- RR mae: the mean absolute error in RR interval detection, i.e., the mean absolute difference between an interval detected by the algorithm and the corresponding reference RR interval.

The values of SDNN ae, RMSSD ae, RR mae are given in milliseconds. Table 1 shows the error values for samples in Welltory-PPG-dataset:

Recall that the TROIKA dataset contains PPG from subjects during treadmill exercises with simultaneous ECG. To verify the accuracy of our algorithm, we chose parts of the PPG data collected in the first 30 s of each PPG signal when the subjects were at rest according to the dataset description. Reference RR intervals were derived from ECG signals using a simple algorithm detecting local maxima of a given minimum height and manually verified for accuracy. Table 2 shows the error values for samples in the TROIKA dataset:

The PPG-DaLiA dataset contains simultaneous PPG and ECG signals during annotated physical activity. For our analysis, we used parts of the signals with activity marked as “sitting”. This gives a signal of approximately 10 minutes for each subject. As in the previous dataset, reference RR intervals were derived from ECG signals using a simple algorithm detecting local maxima of given minimal height and manually verified for accuracy. For each subject, we cut the signal into non-overlapping 100-second segments. For each segment, we applied our algorithm and calculated the discarded ratio, SDNN ae, RMSSD ae, and RR mae metrics. The mean values of the calculated errors per subject are presented in Table 3. Note that the ECG readings for subjects 8 and 12 contain ectopic beats. Those beats were excluded from calculations of reference SDNN and RMSSD values.

## 5. Discussion

### 5.1. Justification of the Ground Truth RR Intervals Source in Welltory-PPG-Dataset

The Polar H10 device was chosen as a source of ground truth labels since it uses ECG for RR interval detection and has demonstrated good accuracy in several studies [38,39,40,41]. In particular, the study [38] reports that the difference between ECG RR intervals and Polar H10 RR intervals has a mean of 0.1 ms and limits of agreement of 2.3 ms at rest, and therefore recommends it as the “gold standard for RR interval assessments”. Chest strap devices were also used as a source of ground truth RR intervals in a number of research papers [42,43]. Additionally, the collected RR intervals in the Welltory-PPG-dataset were manually examined by an expert to ensure their accuracy.

### 5.2. Justification of the Chosen Methods

#### 5.2.1. Signal Preprocessing

We chose the simple preprocessing procedure of Section 3.1 to avoid most basic signal issues such as vanished signal and abrupt shift. Since CWT with Mexican hat wavelet does not require baseline removal [24], this simple preprocessing is sufficient and also allows us to avoid signal distortion with more extensive preprocessing.

#### 5.2.2. Heart Rate Estimation

Non-stationarities in the PPG signals are relatively moderate, as the heart rate changes gradually for healthy individuals at rest. Therefore STFT provides an adequate estimate of the time-frequency presentation of the signal and allows us to estimate the moving average heart rate.

#### 5.2.3. Wavelet Based Peak Detection

We chose the Mexican hat function as the mother wavelet due to its good time–space resolution [44]. Other commonly used wavelets such as Morlet can provide a better resolution in the frequency-space, however, they show a less accurate localization in the time-domain and therefore reduce accuracy in peak detection. Note that the Mexican hat wavelet is broad in frequency, which can create difficulties when CWT is used for time–frequency localization, however, for the task of peak detection, it is more important to provide good localization in the time domain.

The scale range for the CWT from 0.05 to 0.3 is chosen empirically. The smallest scale 0.05 provides enough small-scale details of the initial signal, and decreasing the smallest scale does not give any accuracy improvements of R-peak detection.

### 5.3. Relation to Previous Work

In the heart rate detection part, we estimated heart rate by constructing a continuous curve consisting of peaks in the spectrogram. This approach is similar to one used in [7]. The difference is that in our context we do not expect heavy motion artifacts, as the data are collected at rest, and we do not have access to accelerometer data to filter out the motion impact. However, as we expect our PPG signals to be collected in an uncontrolled environment, we also do not have an initialization period as in [7], where we can safely choose the location of the highest peak in the periodogram as the heart rate estimate. Instead, we use a rough estimate using counting of peaks and zero crossings in a sufficiently smooth approximation to the signal as a prior estimate and then choose peaks in the periodogram that are closest to the prior estimate.

Ridge lines in the CWT with the Mexican hat mother wavelet were also used for peak detection in [24]. The novelty in our approach is that we use the length of the ridge lines as a feature to choose the right peaks, rather than the signal-to-noise ratio used in [24]. Another difference is that in our context we can use almost-periodicity of PPG signals as a heuristic when choosing the ridge lines. Moreover, in our approach we use the similarity in the ridge lines shapes to estimate the PPG signal quality.

### 5.4. Accuracy Comparison with Other Algorithms

Among the variety of algorithms proposed for automatic heart rate variability estimation from PPG [13,14,17,19,20,22] only [20] was tested on publicly available data. The algorithm of [20] was tested on the TROIKA dataset, reporting sufficiently large errors in HRV metric estimations (with SDNN error on average −37.29 and standard deviation of 59.67 ms). However, the test was run on entire signals, including treadmill exercise, where inter-beat intervals derived from PPG (even if detected correctly) cannot be used as substitutes of RR intervals for HRV estimation as shown in [12,15]. Therefore it is not possible to directly compare the results of our algorithm with the results reported in [20].

### 5.5. Limitations

**Figure 11:**
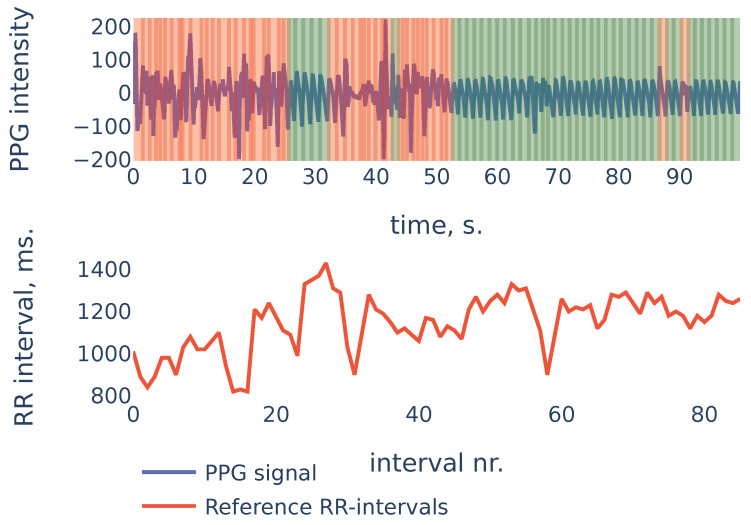
PPG-DaLiA, Subject S3, interval 0 to 100 s.

To give more insight into algorithm accuracy, let us consider a typical example where SDNN of the algorithm-detected RR intervals is slightly different from reference intervals’ SDNN. The signal shown in Figure 11 is the first 100 s segment from subject S3 in the PPG-DaLiAdataset:

In the example of Figure 11 the heart rate was decreasing during the first 20 seconds. At the same time, the first 20 seconds of PPG contained a lot of noise and were discarded by the algorithm (the red region on the plot). As a result, the PPG-derived SDNN of 96.66 shows the estimate of the SDNN during the last 80 seconds of the measurement, rather than the whole signal. The reference RR intervals SDNN during the last 80 seconds are 100.80, and due to the non-stationarity of the signal, it is slightly smaller than the whole signal reference SDNN of 136.98.

This example demonstrates one limitation of our algorithm: as the algorithm detects RR intervals in non-corrupted parts of the PPG signal, in the case when the characteristics change slightly over time (as heart rate in the previous example), the HRV estimation produced by the algorithm will be accurate for the non-corrupted interval but may slightly differ from the overall HRV estimates.

### 5.6. Directions for Future Research

The Empirical Wavelet Transform [45] (EWT) is an adaptive method that was successfully applied for EEG signals [46]. As a direction for future research, it would be interesting to see if EWT can be used instead of CWT to improve algorithm accuracy or execution speed.

## 6. Conclusions

We propose an algorithm that can accurately detect peak-to-peak intervals in PPG signals and identify corrupted parts in the signal where such detection is not possible. These requirements are necessary for the algorithm to be suitable for automatic analysis of PPG signals collected in an uncontrolled environment. The algorithm was tested on three publicly available datasets with PPG signals from different sources. The algorithm is able to recognize corrupted parts of PPG signals and the detected intervals provide accurate estimates for the most common HRV metrics: for SDNN, the mean absolute error is 4.07 ms on the Welltory-PPG-dataset, 6.86 ms on the TROIKA dataset and 7.85 ms on the PPG-DaLiAdataset; for RMSSD, it is 6.32 ms on the Welltory-PPG-dataset, 12.3 ms on the TROIKA dataset and on 6.09 ms on the PPG-DaLiAdataset. Therefore, we conclude that the algorithm performs well on various PPG signals; it can be used to automatically analyze PPG signals obtained in an uncontrolled environment; for PPG collected from healthy subjects at rest, the detected intervals can be used for accurate and reliable estimates of basic HRV metrics. The algorithm cannot be used for HRV estimation from PPG signals collected during or right after exercise since, in that case, the PPG signal does not contain sufficient information [12,15].

## Figures and Tables

**Table 1:** Algorithm performance on the Welltory-PPG-dataset.

Subject	Discarded Ratio	SDNN ae (ms)	RMSSD ae (ms)	RR mae (ms)
01	0.762	3.683	4.058	24.667
02	0.121	2.784	6.977	10.436
03	0.540	4.604	3.802	7.043
04	0.092	6.527	5.242	6.586
05	0.050	2.573	3.161	4.509
06	0.030	1.831	4.913	5.320
07	0.700	11.076	11.658	9.733
08	0.165	2.116	4.702	12.906
09	0.089	1.495	6.863	13.255
10	0.446	8.965	10.778	5.657
11	0.400	9.285	12.619	8.095
12	0.449	0.389	11.739	11.492
13	0.265	4.257	13.563	11.773
14	0.267	4.894	6.743	7.258
15	0.127	1.339	1.795	7.053
16	0.000	5.040	4.900	6.990
17	0.051	1.583	3.145	6.477
18	0.030	0.262	1.854	8.374
19	0.186	6.485	10.311	10.649
20	0.096	0.717	3.753	8.784
21	0.111	5.505	0.192	4.379
**mean**	**0.237**	**4.067**	**6.322**	**9.116**

**Table 2:** Algorithm performance on the TROIKA dataset.

Subject	Discarded Ratio	SDNN ae (ms)	RMSSD ae (ms)	RR mae (ms)
01	0.167	0.489	3.692	7.667
02	0.805	19.851	23.394	10.625
03	0.688	4.258	11.224	6.267
04	0.659	5.721	0.450	9.214
05	0.396	2.786	6.625	5.250
06	0.389	2.199	0.422	10.545
07	0.234	2.184	1.810	9.611
08	0.282	16.938	21.814	15.679
09	0.100	0.631	11.768	9.694
10	0.548	13.896	32.186	19.286
11	0.364	6.520	8.692	8.857
12	0.306	6.897	25.570	14.029
**mean**	**0.411**	**6.864**	**12.304**	**10.560**

**Table 3:** Algorithm performance on the PPG-DaLiA dataset.

Subject	Discarded Ratio	SDNN ae (ms)	RMSSD ae (ms)	RR mae (ms)
01	0.141	5.508	7.831	7.998
02	0.229	7.500	2.791	6.902
03	0.246	16.139	4.506	8.054
04	0.439	9.181	5.910	8.245
05	0.215	2.072	0.595	5.187
06	0.147	2.232	1.217	5.313
07	0.083	9.092	11.238	8.294
08	0.601	19.157	15.353	15.151
09	0.387	4.480	8.267	12.926
10	0.150	3.819	5.406	9.754
11	0.215	5.878	4.894	6.480
12	0.348	18.955	10.483	7.594
13	0.053	4.412	3.153	4.876
14	0.088	3.633	4.514	6.883
15	0.109	5.688	5.175	8.135
**mean**	**0.230**	**7.850**	**6.089**	**8.120**

## Data Availability

The newly introduced Welltory-PPG-dataset is publicly available at https://github.com/Welltory/welltory-ppg-dataset (accessed on 1 July 2021). Other datasets used in this paper are PPG-DaLiA [8] available at https://archive.ics.uci.edu/ml/datasets/PPG-DaLiA (accessed on 1 July 2021) and TROIKA [7] available at https://sites.google.com/site/researchbyzhang/ieeespcup2015 (accessed on 1 July 2021).

## Abbreviations

The following abbreviations are used in this manuscript:

PPG	Photoplethysmography
ECG	Electrocardiography
EEG	Electroencephalography
RR intervals	Intervals between consecutive R peaks
STFT	Short-time Fourier transform
CWT	Continuous wavelet transform
HRV	Heart rate variability
SDNN	Standard deviation of RR intervals, cf. Equation (1)
RMSSD	Root mean square of the successive differences of RR intervals, cf. Equation (2)
MAE	Mean absolute error
